# Advent of extreme events in predator populations

**DOI:** 10.1038/s41598-020-67517-1

**Published:** 2020-06-30

**Authors:** Sudhanshu Shekhar Chaurasia, Umesh Kumar Verma, Sudeshna Sinha

**Affiliations:** 1 Indian Institute of Science Education and Research Mohali, Knowledge City, Sector 81, Manauli, 140306 India; 2 Indian Institute of Technology, Bombay, Powai, Mumbai, 400076 India; 3 Indian Institute of Science Education and Research Tirupati, Tirupati, 517507 India

**Keywords:** Physics, Statistical physics, thermodynamics and nonlinear dynamics

## Abstract

We study the dynamics of a ring of patches with vegetation–prey–predator populations, coupled through interactions of the Lotka–Volterra type. We find that the system yields aperiodic, recurrent and rare explosive bursts of predator density in a few isolated spatial patches from time to time. Further, the global predator biomass also exhibits sudden uncorrelated occurrences of large deviations from the mean as the coupled system evolves. The maximum value of the predator population in a patch, as well as the maximum value of the predator biomass, increases with coupling strength. These trends are further corroborated by fits to Generalized Extreme Value distributions, where the location and scale factor of the distribution increases markedly with coupling strength, indicating the crucial role of coupling interactions in the generation of extreme events. These results indicate how occurrences of extremely large predator populations can emerge in coupled population dynamics, and in a more general context they suggest a generic class of deterministic nonlinear systems that can naturally exhibit extreme events

## Introduction

Due to their huge impact in phenomena that range from traffic jams to weather disturbances, the existence of extreme events has triggered much research interest^1^. An extreme event can be considered as one where a state variable (or variables) in an engineered or natural system exhibits large deviations from the average, i.e. the system is interrupted by sudden excursions to values that are significantly different from the mean value, with such deviations being aperiodic, recurrent and rare. Typically an extreme event can be said to have occurred if a variable is several standard deviations away from the mean, and such unusually large values signal occurrences of catastrophic significance. Examples of such extreme events are found in weather patterns^2^, ocean waves^3^, financial crashes^4^, black-outs in power grid networks^5,6^ and optical systems^7^.

The search for generic mechanisms that naturally yield such extreme events is an issue of vital importance for the basic understanding of complex systems, as well as real world applications^8^. Efforts to obtain extreme events typically involve stochastic models^9,10^, such as a recent random walk model of transport on networks^11^. In the arena of deterministic dynamical systems, there have also been a few recent studies on extreme event generation in coupled systems. Such systems have typically been composed of diffusively coupled individual units that are excitable systems, which are capable of self-generating large deviations^12–14^. It is important however to find broad coupling classes that can provide mechanisms to induce extreme events in dynamical systems that are not capable of generating such extreme events in isolation. Unearthing such deterministic systems would offer non-trivial examples of extreme events arising from interactions^8^, rather than intrinsic or noise-driven large deviations in the states of the constituent systems.

Here we explore the emergence of extreme events in a ring of patches with vegetation–prey–predator populations, coupled through interactions of the Lotka–Volterra type. Unlike many earlier models yielding extreme events, our model has no stochastic environmental influences or sources of random fluctuations, in either the state variables or the parameters determining the dynamics of isolated sites.

Rather, we present a new scenario for the advent of extreme events in both space and time, in a system of populations coupled through generic Lotka–Volterra type interactions, suggesting a generic coupling class that can naturally yield extreme events in interactive deterministic nonlinear systems.

## Coupled population model

In this work we consider a model for the population fluctuation in snowshoe hare and the Canadian lynx, that fits observed data well^15^, consisting of a three-level vertical food chain with predators feeding on herbivores, which in turn consume vegetation. The dynamical equations describing the time evolution of this three-level trophic system composed of interacting vegetation (denoted by *u*), prey (denoted by *v*) and predator (denoted by *w*) populations are given by the following functions *f*(*u*, *v*, *w*), *g*(*u*, *v*, *w*) and *h*(*u*, *v*, *w*):

1 $$\begin{aligned} {\dot{u}}= & {} f(u,v,w) \ = \ a u -\alpha _1 f_1(u,v)\nonumber \\ {\dot{v}}= & {} g(u,v,w) \ = \ -b v+\alpha _1 f_1(u,v)-\alpha _2 f_2(v,w) \nonumber \\ {\dot{w}}= & {} h(u,v,w) \ = \ -c(w-w^\star )+\alpha _2 f_2(v,w) \end{aligned}$$

Here the consumer–resources and predator–prey interactions are modelled using the Holling type II term and the Lotka–Volterra term, described by functions $f_1(u,v)$ and $f_2(v,w)$ respectively. The Holling type II functional form $f_1(u,v) = uv/(1+ku)$ represents the coupling of vegetation (*u*) and prey (*v*) with coupling strength $\alpha _1$. The Lotka–Volterra functionial form $f_2(v,w) = v w$, represents the coupling of prey (*v*) and predator (*w*) with coupling strength $\alpha _2$. The parameters *a*, *b* and *c* represent intrinsic growth rates of trophic species *u*, *v* and *w*. Further the model allows the predator *w* to maintain a low equilibrium level $w = w^{\star }$ even when the population of its usual prey is low. This situation arises when alternative food sources are available for the predator (such as red squirrels for the Canadian lynx in this case) and is mathematically modelled by linearizing the predator growth rate about the equilibrium level as $(w-w^{\star })$. In this work we chose the parameters to be $a=1.0$, $b=1.0$, $c=10.0$, $w^\star =0.006$, $\alpha _1=0.5$, $\alpha _2=1.0$ and $k=0.05$, consistent with empirical data as reported in Ref.^15^.

**Figure 1:**
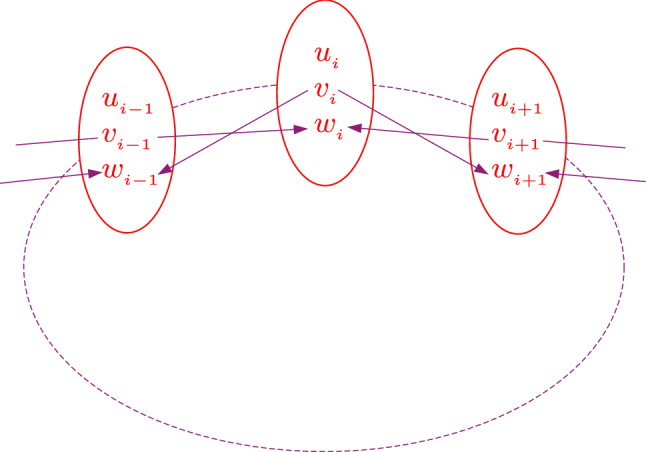
Schematic diagram of the interaction of a patch with nearest neighbour patches, in a ring of population patches. The system has periodic boundary conditions.

Now we expand the scope of the model above to mimic a collection of such population patches, consisting of vegetation, preys and predators. The populations in a local patch interact with other nearby patches in such a way that the predator of one patch can attack prey in neighbouring patches, namely the patches are coupled via cross-predation. Figure 1 shows a schematic diagram of the interaction of a patch with nearby patches. Specifically, we consider a ring of such patches, with each patch indexed spatially by *i*, $i=1,\dots N$, where *N* is the total number of patches in the ring. The populations of vegetation, prey and predator in patch *i* is represented by $u_i$, $v_i$ and $w_i$ respectively.

The form of the predator–prey interaction between nearest neighbouring patches is of the Lotka–Volterra type and is given by the following set of dynamical equations:

2 $$\begin{aligned} \dot{u_i}= & {} f(u_i,v_i,w_i) \nonumber \\ \dot{v_i}= & {} g(u_i,v_i,w_i) - \ \frac{C}{2} \ \{ \ v_i w_{i-1} + v_i w_{i+1} \}\nonumber \\ \dot{w_i}= & {} h(u_i,v_i,w_i) + \ \frac{C}{2} \ \{ w_i v_{i-1} + w_i v_{i+1} \} \end{aligned}$$

The coupling constant *C* reflects the strength of interaction among patches, and in this work we focus on this crucial parameter. Note that in formal terms this constitues a conjugately-coupled system^16^.

So the scenario this model captures is the following: predators are usually more mobile and can reach other patches to find prey. So the predators of neighbouring patches influence the population of prey, as the prey (for instance, the snowshoe hare in this particular example) can be devoured by predators (the Candian lynx in this example) of neighbouring patches. As a consequence the population of predators depends on the population of prey of the neighbouring patches, as they can make forays into neighbouring areas to consume prey. This scenario is modelled by the inter-patch coupling in the standard Lotka–Volterra form.

## Spatial distribution and temporal evolution of the population densities in the network

The first quantity of relevance in this system is the *local population density* in the patches, and their temporal fluctuations. We will focus on the deviation of the local population densities from the mean value, i.e., we will look for the emergence of extreme events in the local patches, as evident in explosive population densities at specific spatial locations.

**Figure 2:**
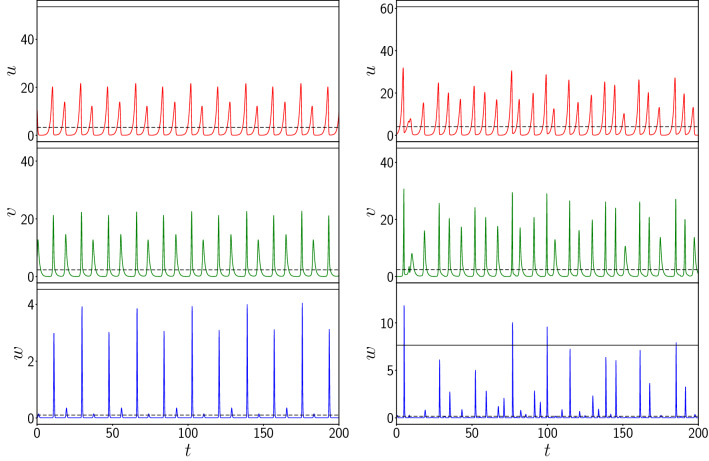
Time evolution of the vegetation *u*, prey *v* and predator *w* populations of one representative patch, from random initial states, for the following cases: (left) uncoupled patches and (right) patches coupled to neighbouring patches, with coupling strength $C=1.0$ in Eq. (2). The black dashed and black solid lines represent the mean ($\mu$) and ten times the standard deviation $\sigma$ from the mean (i.e. $\mu +10\sigma$) respectively.

**Figure 3:**
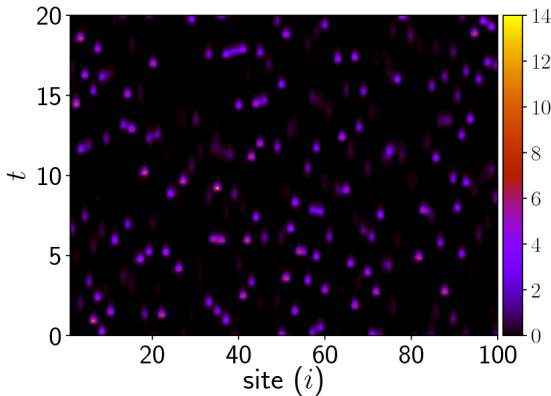
Space-time density plot of the predator population $w_i(t)$ at site $i=1, \dots 100$, for the case of patches coupled to neighbouring patches, with coupling strength $C=1.0$ in Eq. (2).

Figure 2 shows the time evolution of the vegetation, prey and predator populations of one representative patch from the network at high coupling strength $C=1.0$. We observe that, while the population densities are mostly confined to low values, the evolution is punctuated by sudden boosts to very high values. For instance, local predator population densities can shoot up more than 10 standard deviations away from the mean value. This is evident in the lower right panel of Fig. 2, where one can see instances where *w* exceeds the $10 \sigma$ threshold. The instants at which these large fluctuations occur are relatively rare and completely *uncorrelated* in time and space. These large deviations are also clearly evident through the space-time plot of the evolution of predator populations in different patches in the network, shown in Fig. 3, where the extreme values of predator populations are visible as sparse bright randomly located dots in the figure.

Note that if consider a lower threshold (e.g. $\mu + 4 \sigma$), the vegetation, prey and predator all exhibit events that exceed the threshold, with prey and predator populations yielding above-threshold events even when uncoupled. However significantly, such occurrences in uncoupled patches are strictly *periodic*, and stem from the intrinsic pulse-like solutions of the constituent patches.

**Figure 4:**
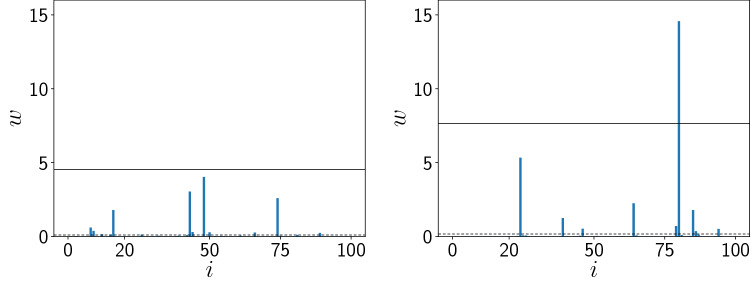
Spatial distribution of the predator population $w_i$ ($i=1, \dots 100$), at a representative instant of time, for the following cases: (left) uncoupled patches, and (right) patches coupled to neighbouring patches, with coupling strength $C=1.0$ in Eq. (2). The black dashed and black solid lines represent the mean ($\mu$) and ten times the standard deviation $\sigma$ from the mean (i.e. $\mu +10\sigma$) respectively. Note that the predator population $w_i$ in the most patches, as well as the spatial average of *w* at an instant of time, is so low that it is barely visible in the figures.

In Fig. 4 we display the spatial distributions of the predator populations $w_i$ for the patches $i=1, \dots N$ at a representative instant of time, for the case of uncoupled patches and coupled patches. It is clearly seen that there are a few patches in the coupled case that grow explosively and have a predator population much larger than the mean value. These results qualitatively suggest that the coupling of population patches give rise to extreme predator populations at a small number of spatial locations, analogous to an *extreme event in space.* Such catastrophic events are rare in space, and occurs only at a couple of sites, but signal significant damage as they may entail serious control costs.

## Global maximum of predator populations

Now we quantitatively estimate the maximum vegetation, prey and predator population densities in a patch, denoted by $u_{max}$, $v_{max}$ and $w_{max}$ respectively, attained in the course of the network dynamics^17,18^. We estimate this by finding the global maximum of $u_i$, $v_i$ and $w_i$, for $i=1, \dots N$ (where *N* is sufficiently large), sampled over a time interval *T* (where *T* is much longer than the intrinsic oscillation period). This will help us gauge the magnitude of the extreme event and its relation to coupling strengths. Here we present results with $T=50$, with no loss of generality.

**Figure 5:**
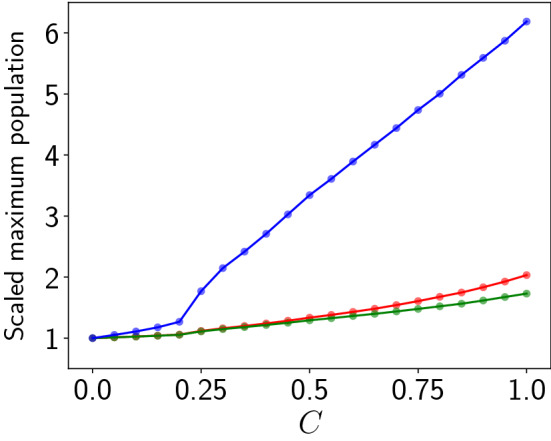
Dependence of the global maximum of the vegetation population $u_{max}$ (red), the prey population $v_{max}$ (green) and the predator population $w_{max}$ (blue), occurring in the patches in an interval of time $T=50$, on coupling strength, for $N=100$. Here we depict the *scaled* values of the maximum, where $u_{max}$, $v_{max}$ and $w_{max}$ are divided by their values in the uncoupled (i.e. $C=0$) case. Note that larger systems yield larger $u_{max}$, $v_{max}$ and $w_{max}$, with the values saturating to a characteristic value in the limit of large system size.

Figure 5 shows the maximum $u_{max}$, $v_{max}$ and $w_{max}$, for a wide range of coupling strengths of the population patches. In the figure we depict the *scaled* values of the maxima, where $u_{max}$, $v_{max}$ and $w_{max}$ are divided by their values in the uncoupled case. These scaled quantities help us assess the increase in the maxima in coupled networks, compared to that obtained in uncoupled patches, allowing us to specifically gauge the coupling-induced effects on the emergent maximum population densities. It is evident from the simulation results that the magnitude of the maximum vegetation and prey populations (i.e. $u_{max}$ and $v_{max}$ denoted by the red and green curves) do not increase much as coupling strength increases. However, the maximum predator population increases very significantly with coupling strength, with $w_{max}$ at $C=1$ exceeding over six-fold the value obtained for uncoupled patches.

Further, we also explored different $\alpha _2$ values in Eq. (1), reflecting different intra-patch predation rates, where the dynamics of the uncoupled system exhibited very low peaks in the population densities, specifically an *order of magintude* smaller than that occurring in the uncoupled system displayed in Fig. 2. Interestingly, there too we observed clear evidence of extreme events in predator populations arising under coupling. This suggests that the presence of significant peaks in the coupled system is not necessary for the advent of extreme events.

Additionally note that increasing the strength of the predator–prey interaction in a single patch does not give rise to extreme events in the population dynamics. Rather, as this coupling strength increases, the maximum of the predator population decreases and the dynamics becomes more regular. This lends further support to the importance of inter-patch coupling in the generation of large deviations in predator populations.

## Temporal evolution of biomass

The next quantity of interest is the total biomass of the vegetation, prey and predators, denoted by $b_u$, $b_v$ and $b_w$ respectively. This represents a *collective dynamical quantity*, and is given at an instant of time *t* as follows:

3 $$\begin{aligned} b_u (t) = \sum _{i=1}^N u_{i}(t) \ , \ b_v (t) = \sum _{i=1}^N v_{i}(t) \ , \ b_w (t) = \sum _{i=1}^N w_{i}(t) \end{aligned}$$

where *N* is the system size. Again, we will examine the presence of large excursions from mean-values, as such explosive growth indicate extreme events in time of a global quantity.

**Figure 6:**
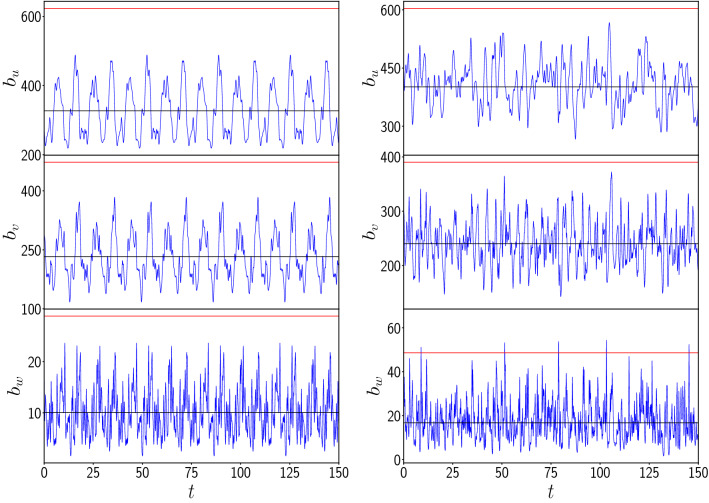
Time evolution of the biomass $b_u (t)$, $b_v (t)$ and $b_w (t)$ of the vegetation, prey and predator populations respectively, from random initial states, for the following cases: (left) uncoupled patches and (right) patches coupled to neighbouring patches, with coupling strength $C=1.0$ in Eq. (2). The black and red lines represent the mean ($\mu$) and four times the standard deviation $\sigma$ from the mean (i.e. $\mu +4\sigma$) respectively.

**Figure 7:**
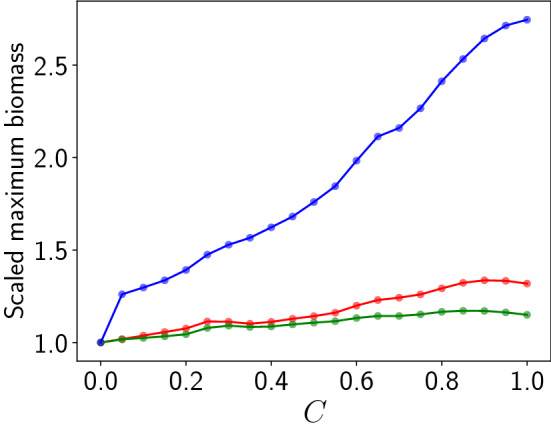
Dependence of the maximum of vegetation biomass $b_u$ (red), the prey biomass $b_v$ (green) and the predator biomass $b_w$ (blue), in an interval of time $T=50$, on coupling strength, for $N=100$. Here we depict the values of the maximum scaled by their values in the uncoupled case.

Figure 6 shows the biomass of the vegetation ($b_u$), prey ($b_v$) and predators ($b_w$). The uncoupled case is shown alongside as a reference. It is clear that when the patches are uncoupled, $b_u$, $b_v$ and $b_w$ do not experience any large fluctuations. Further, the biomass of vegetation and prey for the coupled case also stays bounded within the $4 \sigma$ threshold. However, interestingly, the predator biomass in coupled patches occasionally builds up to extreme values, crossing the $4 \sigma$ threshold. This is also corroborated through a comparison of the maximum values of the biomass of the vegetation, prey and predator in the coupled network vis-a-vis the uncoupled patches. The maximum prey biomass is almost unchanged on coupling, and the maximum vegetation biomass in a coupled network exhibits less than $20 \%$ change from uncoupled values. On the other hand, the maximum predator biomass in a coupled network exhibits a *three-fold* increase compared to uncoupled patches (cf. Fig. 7). So coupling has a very significant effect on the predator biomass, and one finds clear evidence of the emergence of extreme events in time for this global quantity.

**Figure 8:**
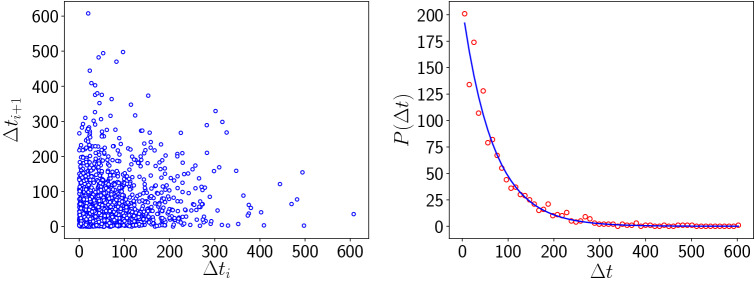
(Left) Return map of $\Delta t_{i+1}$ vs $\Delta t_i$, and (right) Probability distribution of $\Delta t_i$ fitted with exponentially decaying function, where $\Delta t_i$ is the *i*th interval between successive extreme events, where an extreme event is defined at the instant when biomass crosses the $\mu +4\sigma$ line (cf. Fig. 6). Here the system size $N=100$ and coupling strength $C=1.0$ in Eq. (2).

In order to ascertain that the extreme values are uncorrelated and aperiodic we examine the time intervals between successive extreme events in the predator biomass evolution. Figure 8 shows the return map of the intervals between extreme events and it is clearly shows no regularity. The probability distribution of the intervals is also Poisson distributed and so the extreme predator population buildups are uncorrelated aperiodic events.

Also note that spatial heterogeneity plays a role in the emergence of extreme events. If one considers a homogeneous system, the biomass does not exhibit any large deviations from the mean. Only a system comprised of a spatially inhomogeneous distribution of vegetation, predator and prey, yields extreme values of the global biomass. This suggests the importance of spatial heterogeneity in the sudden emergence of large biomass from time to time.

## Generalized extreme value distribution

Lastly we analyse the distribution of the maximum size of the predator populations, as well as the maximum size of the global predator biomass. In probability theory and statistics, the generalized extreme value (GEV) distribution is a family of continuous probability distributions developed within extreme value theory, and the GEV distribution is often used as an approximation to model the maxima of long finite sequences of random variables^19^. So in order to obtain a quantitative measure of the extreme events generated for different coupling strengths, we fit these probability distributions to the GEV distribution, given by:

$$\begin{aligned} f(y;\zeta )= {\left\{ \begin{array}{ll} \exp (-(1-\zeta y)^{1/\zeta })(1-\zeta y)^{(1/\zeta ) -1},&{} \zeta >0,\\ \ &{} \ y\le 1/\zeta \\ \exp (-\exp (-y))\exp (-y), &{} \zeta =0 \end{array}\right. } \end{aligned}$$

with

$$\begin{aligned} y(x,\mu ,\sigma )= & {} (x-\mu )/\sigma \end{aligned}$$

**Figure 9:**
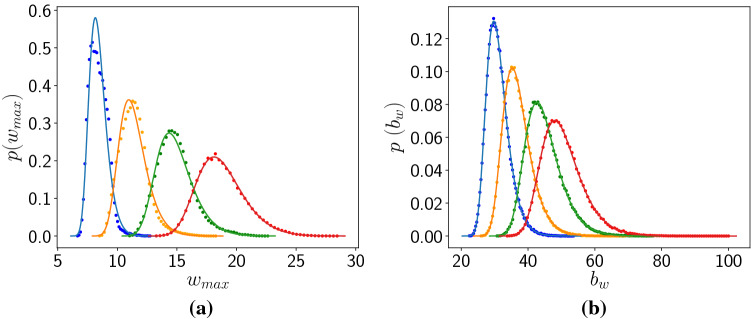
Probability distribution of the (**a**) maximum predator population in a patch $w_{max}$, and (**b**) maximum predator biomass, obtained from sampling the predator populations in a time interval $T=50$ (with no loss of generality). The fit of the data from numerical simulations to the Generalized Extreme Value distribution is shown by solid lines, for coupling strength $C=0.4$ (blue), 0.6 (orange), 0.8 (green) and 1.0 (red).

Here $\zeta$ is the shape parameter, $\mu$ is the location parameter and $\sigma$ is the scale parameter. Note that the scale parameter is the most relevant parameter in this case as it determines the spread of the distribution (see Fig. 9). So larger scale parameters indicate larger deviations from the mean.

**Figure 10:**
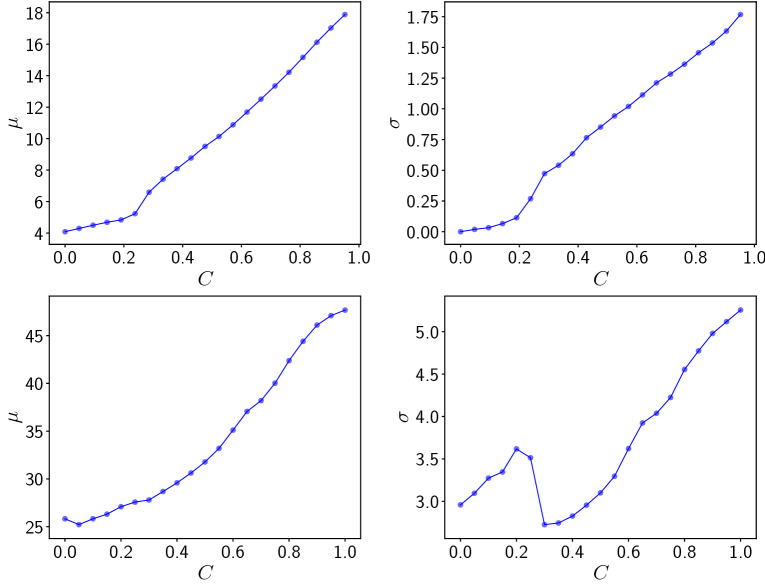
Location (left) and scale (right) parameters obtained by best-fit to the Generalized Extreme Value distribution of (top) maximum predator population in a patch $w_{max}$, and (bottom) maximum predator biomass $b_w$, obtained from sampling a time interval of $T=50$ (cf. Fig. 9), for different coupling strengths *C*.

Figure 10 shows the location and scale parameters obtained by best-fit to the Generalized Extreme Value distribution of the maximum predator population in a patch $w_{max}$, and the maximum predator biomass $b_w$. Clearly the location and scale parameters increase monotonically with coupling strength, for coupling strengths higher than $\sim 0.4$, with the rise being approximately linear at high *C*. Increasing location parameters indicate that the average predator population in a patch and the average predator biomass increase almost linearly with coupling strength. Increasing scale parameters suggest that the distribution becomes increasingly spread out, and the tail of the distribution extends to larger values. So more extreme predator populations can be expected to occur in the patches, from time to time, when the coupling between the patches is stronger.

## Effect of increasing range of coupling

Finally, we have also explored the effect of increasing spatial range of interactions, with predator–prey interactions occuring over 2*k* neighbouring patches, given by the following set of dynamical equations:

4 $$\begin{aligned} \dot{u_i}= & {} f(u_i,v_i,w_i) \nonumber \\ \dot{v_i}= & {} g(u_i,v_i,w_i) - \ \frac{C}{2k} \sum _{j=1}^k \ \{ \ v_i w_{i-j} + v_i w_{i+j} \}\nonumber \\ \dot{w_i}= & {} h(u_i,v_i,w_i) + \ \frac{C}{2k} \sum _{j=1}^k \ \{ w_i v_{i-j} + w_i v_{i+j} \} \end{aligned}$$

Here *k* reflects the spatial range of the coupling, and determines the number of neighbouring patches over which the prey-predator interactions occur. Specifically the coupling range $2k=k^{\prime }$, where $k^{\prime }$ is the degree of the underlying ring connection network. The case of nearest neighbour interactions considered earlier is the limiting case of this model with $k=1$. Also note that for a homogeneous system, the inter-patch coupling term is the same for all *k*, as the system reduces to a set of identical population patches given by dynamical equations where the second and third equations in Eqns. 1 are simply augmented by the product of variables *v* and *w* weighted by the coupling constant *C*.

**Figure 11:**
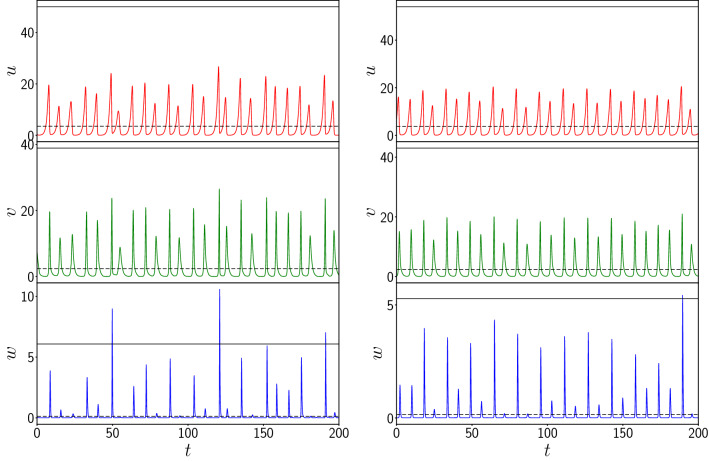
Time evolution of the vegetation *u*, prey *v* and predator *w* populations of one representative patch, from random initial states, for the following cases: (left) patches coupled to 4 neighbouring patches, and (right) patches coupled to 50 neighbouring patches, with coupling strength $C=1.0$ in Eq. (4). The black dashed and black solid lines represent the mean ($\mu$) and ten times the standard deviation $\sigma$ from the mean (i.e. $\mu +10\sigma$) respectively. Clearly the vegetation and prey populations do not exhibit large deviations from the mean, while the predator population occasionally exceeds the $10 \sigma$ threshold.

Figure 11 shows the time evolution of the vegetation, prey and predator populations of one representative patch from the network at high coupling strength $C=1.0$ for different spatial ranges of coupling interactions. It is evident that while the population densities are mostly confined to low values, the evolution of the predator population is punctuated by sudden boosts to more than 10 standard deviations away from the mean value, for the case of coupling to 4 nearest neighbour patches (cf. lower left panel of Fig. 11). Interesting however, these large population buildups are less intense when coupling occurs over a larger spatial range (cf. right panel of Fig. 11). So, while increasing coupling strength monotonically increases the occurence of extreme events (cf. Fig. 5), large deviations are more enhanced when the spatial range of coupling is smaller, i.e. strong interactions over shorter ranges give rise to more extreme deviations from mean values.

**Figure 12:**
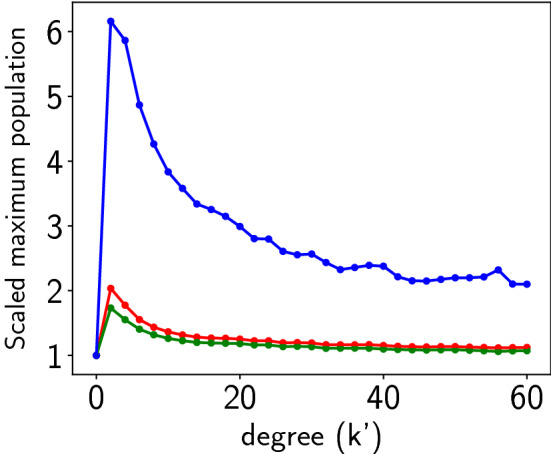
Dependence of the global maximum of the vegetation population $u_{max}$ (red), the prey population $v_{max}$ (green) and the predator population $w_{max}$ (blue), occurring in the patches in an interval of time $T=50$, on coupling range $2k=k^{\prime }$, where $k^{\prime }$ is the degree of the underlying ring connection network, for system size $N=100$ and coupling strength $C=1$. Here we depict the *scaled* values of the maximum, where $u_{max}$, $v_{max}$ and $w_{max}$ are divided by their values in the uncoupled (i.e. $C=0$) case.

Figure 12 shows the maximum $u_{max}$, $v_{max}$ and $w_{max}$, with respect to coupling range, varying from nearest neighbour connections to near global coupling. As before, in the figure we depict the *scaled* values of the maxima, where $u_{max}$, $v_{max}$ and $w_{max}$ are divided by their values in the uncoupled case, as these scaled quantities help us assess the increase in the maxima in coupled networks, compared to that obtained in uncoupled patches, allowing us to specifically gauge the coupling-induced effects on the emergent maximum population densities. It is evident from the simulation results that the magnitude of the maximum vegetation and prey populations (i.e. $u_{max}$ and $v_{max}$ denoted by the red and green curves) do not increase much as coupling range increases. However, the maximum predator population increases very significantly with coupling range, with $w_{max}$ at $k^{\prime }=2$ exceeding over 6-fold the value obtained for uncoupled patches. However again, as noticed earlier, unlike the effect of increasing coupling strength (cf. Fig. 5), increasing coupling range does not enhance the maximum value monotonically. Rather, the extreme values are most pronounced for nearest neighbour coupling (i.e. $k^{\prime }=2$). As coupling range further increases, the maxima saturate to a value approximately twice that of the uncoupled case. Similar trends are also observed for the biomass of vegetation, prey and predator for varing coupling ranges.

So we find that increasing the spatial range of coupling does not lead to monotonic enhancement of extreme events. Rather, there is an optimal size of coupling neighbourhood that maximizes the effect, and increasing coupling neighbourhoods further leads to reduction of the magnitude of extreme events. This indicates that the advent of large deviations arise from a subtle and non-trivial interplay of coupling range and coupling strength, and largest deviations from mean values emerge when there are strong interactions over small number of neighbouring patches.

## Conclusions

In summary, we have studied the population dynamics of a ring of patches with vegetation, preys and predators, where the patches are coupled through interactions of the Lotka–Volterra type. We find that this system yields extreme events in the predator population in the patches, with bursts of explosive predator population growth in a few isolated patches from time to time. Further, the global predator biomass also yields extreme values as the coupled system evolves. The maximum value of the predator population in a patch, as well as the maximum value of the predator biomass, *increases with coupling strength*. Fits of the data from numerical simulations to Generalized Extreme Value distributions also quantitatively corroborate these trends. These results demonstrate the central finding of this work: the Lotka–Volterra class of coupling aids the emergence of temporal and spatial extreme events, indicating a generic mechanism for the occurence of such events in large interactive systems.

Our results then are important, both from the view-point of general models of coupled nonlinear systems, as well as for the more specific implications it may potentially hold for population dynamics. So first we have demonstrated how a *deterministic system, with generic Lotka–Volterra type of interactions, can give rise to extreme events in space and time*. Such examples are uncommon, and so they are significant in the context of general complex systems. Secondly, in the specific context of population dynamics, our model system suggests how predator population densities can grow explosively in certain patches. Though relatively rare, the magnitude of these extreme bursts of predator population density is so huge, that the damage or ensuing cost to control the event, is considerable. Further the biomass of predators can also grow extremely large at certain points in time, and this is of significance due to the catastrophic effects large predator populations can have on the ecosystem as a whole. Lastly, interestingly, the Lotka–Volterra class of interactions has also been used extensively in economic theory^20^, and so our results may have some bearing on extreme events in the financial context.

## References

[1] Albeverio S, Jentsch V, Kantz H (2006). Extreme Events in Nature and Society.

[2] Lubchenco J, Karl T (2012). R. extreme weather events. Phys. Today.

[3] Dysthe K, Krogstad HE, Müller P (2008). Oceanic rogue waves. Annu. Rev. Fluid Mech..

[4] Lillo F, Mantegna RN (2003). Power-law relaxation in a complex system: Omori law after a financial market crash. Phys. Rev. E.

[5] Kinney R, Crucitti P, Albert R, Latora V (2005). Modeling cascading failures in the north american power grid. Eur. Phys. J. B Condens. Matter Complex Syst..

[6] Strogatz, S. How the blackout came to life. *The New York Times* Op. Ed., (25 August 2003).

[7] Solli DR, Ropers C, Koonath P, Jalali B (2007). Optical rogue waves. Nature.

[8] Moitra P, Sinha S (2019). Emergence of extreme events in networks of parametrically coupled chaotic populations. Chaos Interdiscip. J. Nonlinear Sci..

[9] Majumdar SN, Ziff RM (2008). Universal record statistics of random walks and lévy flights. Phys. Rev. Lett..

[10] Schehr G, Majumdar SN (2012). Universal order statistics of random walks. Phys. Rev. Lett..

[11] Kishore V, Santhanam M, Amritkar R (2011). Extreme events on complex networks. Phys. Rev. Lett..

[12] Ansmann G, Karnatak R, Lehnertz K, Feudel U (2013). Extreme events in excitable systems and mechanisms of their generation. Phys. Rev. E.

[13] Karnatak R, Ansmann G, Feudel U, Lehnertz K (2014). Route to extreme events in excitable systems. Phys. Rev. E.

[14] Kingston SL, Thamilmaran K, Pal P, Feudel U, Dana SK (2017). Extreme events in the forced liénard system. Phys. Rev. E.

[15] Blasius B, Huppert A, Stone L (1999). Complex dynamics and phase synchronization in spatially extended ecological systems. Nature.

[16] Karnatak R, Ramaswamy R, Feudel U (2014). Conjugate coupling in ecosystems: cross-predation stabilizes food webs. Chaos Solit. Fract..

[17] Balakrishnan V, Nicolis C, Nicolis G (1995). Extreme value distributions in chaotic dynamics. J. Stat. Phys..

[18] Nicolis C, Balakrishnan V, Nicolis G (2006). Extreme events in deterministic dynamical systems. Phys. Rev. Lett..

[19] Coles S (2001). An Introduction to Statistical Modeling of Extreme Values.

[20] Goodwin, R. M. A growth cycle. In *Essays in Economic Dynamics* (Palgrave Macmillan, London, 1982).

